# In-Vehicle Tobacco Smoke Exposure: A Narrative Review of the Literature

**DOI:** 10.3390/ijerph22050658

**Published:** 2025-04-22

**Authors:** Cara Harris, Karen Heaton

**Affiliations:** School of Nursing, The University of Alabama at Birmingham, Birmingham, AL 35294-1210, USA; kharnp@uab.edu

**Keywords:** tobacco smoke, second-hand smoke, smoking cessation, nicotine, motor vehicles, tobacco smoke exposure

## Abstract

(1) Background: This narrative review examines in-vehicle tobacco smoke exposure among private, occupational, and commercial drivers, focusing on prevalence, nicotine biomarkers, and health consequences. (2) Methods: A comprehensive search on the PubMed, Scopus, and Embase databases was used to identify peer-reviewed, full-text, and English articles published between 2014 and 2024. Search terms were related to motor vehicles, tobacco smoke exposure, and drivers. Articles were selected for inclusion based on their relevance to in-vehicle smoking and second- or third-hand smoke exposure attributable to tobacco cigarettes through article title, abstract, and full-text screening. (3) Results: This review highlights the dangers of in-vehicle second- or third-hand smoke exposure, evidenced by the 17 articles included. Significant second-hand smoke exposure and biomarkers were revealed mostly among adolescents and children. However, a gap exists in addressing tobacco smoke exposure among occupational/commercial drivers, specifically, long-haul truck drivers (LHTDs), who have heightened exposure due to their work environment—the truck cabin—which may increase their lung cancer risk. (4) Conclusions: There is a significant literature gap regarding in-vehicle tobacco smoke exposure in occupational/commercial drivers. Future research should include nicotine biomarker usage to quantify nicotine exposures and smoking cessation intervention development tailored to LHTDs.

## 1. Introduction

Cigarette smoking, or first-hand smoke (FHS) exposure, represents the predominant form of tobacco smoke exposure worldwide [1,2]. Cigarette smoking is also both the number one cause of lung cancer [3] and the leading cause of avoidable illness, mortality, and disability in the United States [3]. Cigarette smoking is a global issue significantly contributing to increased cardiovascular mortality [4] and elevated risks of lung cancer [5]. Second-hand smoke (SHS), arising from exposure to the active burning of tobacco through involuntary smoking, poses a grave health risk with no established safe exposure level [5,6]. Globally, SHS exposure affects 34% of non-smoking adults, leading to approximately 603,000 deaths attributed to lung cancer, ischemic heart disease, asthma, and respiratory infections [7]. The United States Environmental Protection Agency (EPA) classifies SHS exposure as a Group 1 carcinogen [8], establishing its carcinogenic effect in humans.

In addition to SHS exposure, the phenomenon of third-hand smoke (THS) exposure further complicates the understanding of tobacco smoke exposure. Third-hand smoke exposure encompasses the involuntary ingestion, inhalation, or absorption of indoor pollutants that remain dormant on surfaces months after tobacco combustion [9]. Despite the widespread presence of THS exposure, its long-term effects remain unknown and not only pose a potential hazard to non-smokers but may also increase carcinogen exposure in smokers [10,11,12]. The unknown effects of THS exposure underscore the need for continued research to unravel its implications and potential public health risks.

Effective indoor smoke elimination is a pivotal measure to prevent non-smokers’ exposure to SHS; nonetheless, SHS exposure continues to pose a significant threat in confined spaces, particularly within vehicles [13]. Beyond tobacco smoke exposure, the existing literature highlights the heightened concentration of other carcinogens, such as polycyclic aromatic hydrocarbons (PAHs), reaching 1325.1 ng/m^3^ (nanograms per cubic meter) in vehicles rather than 16–350 ng/m^3^ when compared to other private settings such as homes [13]. Smoking in a vehicle leaves residual particles on its interior surfaces, which have reactivation capabilities, leading to potential THS exposure for individuals who encounter the contaminated environment [9].

Although tobacco smoke exposure in vehicles is a well-known concept, the lack of a comprehensive literature review over the past ten years extending to occupational and commercial drivers highlights a significant research gap. This gap raises the following question: What is the current state of science related to in-vehicle smoking and nicotine exposure among occupational and commercial drivers? To address this, the original objective of this narrative review was to thoroughly examine the available literature on tobacco smoke exposure among drivers of occupational and commercial drivers; however, this objective was broadened to include all vehicles—private, occupational, and commercial vehicles—due to the gap in the literature focused on occupational and commercial drivers. This review aims to synthesize existing knowledge and create a foundation for future research. Additionally, it may lead to the development of smoking cessation efforts tailored specifically to the trucking community, potentially reducing healthcare costs associated with smoking-related diseases, such as lung cancer and cardiovascular disease. Ultimately, this review will shed light on the harmful effects of continuous smoking in enclosed spaces.

### 1.1. Epidemiological Basis and Concepts of Interest

#### 1.1.1. Lung Cancer

Lung cancer is one of the most common forms of cancer and a leading cause of mortality worldwide [14,15]. Based on cases from 2017 to 2021 and deaths occurring between 2018 and 2022, the prevalence of new lung cancer cases among men and women was 49.0 per 100,000, with a death rate of 32.4 per 100,000 [16]. As of 2021, 610,816 people were living with lung cancer in the United States [16]. Lung cancer is classified into two main types: non-small-cell lung cancer (NSCLC) and small-cell lung cancer (SCLC). Non-small-cell lung cancer further divides into three primary subtypes: adenocarcinoma, squamous-cell carcinoma, and large-cell carcinoma [6]. Lung cancer risk factors include exposure to radon, nicotine, air pollution, and occupational exposures such as asbestos, diesel engine exhaust, arsenic, and chromium, among others [15].

Occupational and commercial drivers such as bus, truck, and taxi drivers have a 28% increased incidence of lung cancer when compared to the general population, based on a study conducted in Korea (SIR 1.28, 95% CI 1.15–1.43) [17]. In the United States, smoking prevalence among occupational drivers (34%) also exceeds that of the general population (25%) [2]. Among long-haul truck drivers (LHTDs) in the United States, 51% are smokers, compared to 19% in the general population [2,18,19], contributing to a concerning 23% increased risk of lung cancer among truck drivers in Northern Italy, specifically, compared to other at-risk populations [14].

While occupational and commercial drivers have an increased risk of lung cancer, non-occupational vehicle passengers also face heightened vulnerabilities. According to the Centers for Disease Control and Prevention, an estimated 10% to 20% of lung cancer cases in the United States—approximately 7300 cases annually—are attributable to SHS exposure in non-smokers [5]. Research further indicates that children are among the most affected populations due to their underdeveloped airways, lungs, and immune systems, as well as their inability to voluntarily avoid exposures [3].

#### 1.1.2. Tobacco Smoke

Conceptually, nicotine exposure levels are primarily derived from tobacco smoke, resulting from the combustion of tobacco leaves. Tobacco smoke use and addiction stem from the nicotine component, commonly measured using nicotine and cotinine biomarkers [20], which can be measured in urine, blood, saliva, hair, or nails [20]. Cigarette smoking leads to smokers’ exposure to many toxins, including ammonia, formaldehyde, carbon monoxide, hydrogen cyanide, nicotine, PAHs, and other carcinogens [20]. The combustion of tobacco, resulting in smoke that is exhaled by smokers and later inhaled by non-smokers, is considered SHS exposure [21]. Second-hand smoke exposure can lead to asthma, acute and chronic respiratory symptoms and conditions, lung cancer, and ischemic heart disease [6,7,21]. Second-hand smoke exposure remains highly concentrated in confined spaces such as vehicles [13]. Exposure to SHS can be detrimental as there is no safe exposure dose level [6]. Indoor smoke elimination is the only way to prevent non-smokers from exposure to SHS. Third-hand smoke exposure includes involuntarily ingesting, inhaling, or absorbing indoor pollutants [9]. The long-term effects of THS exposure are unknown and pose a potential hazard to non-smokers and smokers alike [10,11,12].

Besides tobacco smoke exposure, carcinogens such as PAHs are more concentrated in vehicles (1325.1 ng/m^3^) when compared to other settings (16–350 ng/m^3^) such as homes [13]. Considering the carcinogenic effects of nicotine and other products of tobacco combustion among cigarette smokers [22] and, specifically, the increased concentration of nicotine in the enclosed space of vehicles [13,23], it is clear that a deeper understanding of the state of the science of nicotine exposure in occupational and commercial vehicles is necessary.

#### 1.1.3. Smoking Among Long-Haul Truck Drivers

To cope with stress, fatigue, and boredom during long journeys, LHTDs often use tobacco cigarettes and subsequently become nicotine-dependent [1]. In the United States, cigarette smoking prevalence is more than twice as likely in this population (51%) than in the general public (19%) [2,18,19]. A study conducted in the United States also found that LHTDs are involuntarily exposed to SHS during team driving, where their driving partner engages in cigarette smoking, or during interactions with other smokers [24]. They may experience THS exposure through the reactivation of smoke particles in trucks previously owned by drivers who self-identify as smokers. This exposure occurs when contact is made with surfaces and fabrics, e.g., which are penetrated by cigarette smoke. The enclosed nature of a truck cabin amplifies tobacco smoke exposure, increasing the dose of nicotine exposure and subsequent morbidity and mortality [13,23,25].

## 2. Materials and Methods

An English-language review was conducted to analyze the current literature on occupational and commercial vehicle tobacco smoke exposure prevalence. A literature search was conducted across multiple databases, including PubMed, Scopus, and Embase. Inclusion criteria included manuscripts related to in-vehicle smoking, SHS or THS exposure attributable to tobacco cigarette combustion in motor vehicles, or biomarkers of tobacco smoke in vehicles. Initially, studies exclusively related to the combined exposure for both homes and vehicles were excluded; however, prior to full-text screening, the inclusion criteria were revised to encompass both settings, aiming to enhance the breadth of articles supporting vehicle exposure. All previously screened abstracts were reassessed under the revised criteria to minimize the risk of excluding relevant references and potential selection bias. Articles evaluating the impacts of smoking ban legislation and its impacts, studies solely focused on SHS exposure caused by e-cigarettes, and studies concentrating solely on SHS exposure in homes were excluded. Articles addressing SHS exposure in settings other than vehicles or homes were also excluded.

**Figure 1:**
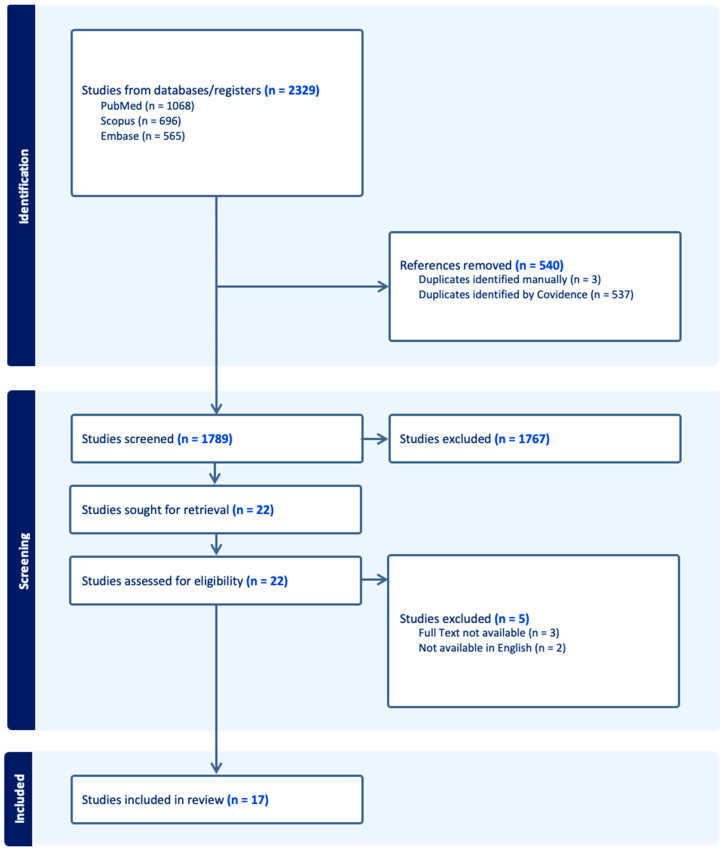
Preferred Reporting Items for Systematic Reviews and Meta Analysis (PRISMA) Diagram.

The articles were available in full text, were written in English, and were published from 2014 to 2024. An initial search using the terms described in Table 1 yielded 1068 PubMed, 696 Scopus, and 565 Embase results. The combined results yielded 2329 articles. After 540 duplicates were removed, 802 studies were screened for eligibility. Of the 1789 manuscripts screened, 1767 studies were excluded, leaving 22 manuscripts for consideration. Finally, 2 of the 22 articles were later excluded for lack of availability in the English language, and 3 articles were excluded for no full-text availability, leaving 17 for inclusion in this review (Figure 1).

## 3. Results

The literature search for tobacco smoke exposure in vehicles resulted in the identification of seventeen articles [8,26,27,28,29,30,31,32,33,34,35,36,37,38,39,40]. All articles collectively revealed a significant prevalence of SHS exposure in vehicles, highlighting its associated risks and subsequent health impacts. However, of the available literature, none of the seventeen studies reviewed focused on commercial or occupational drivers; instead, they primarily included children, adolescents, and adults as members of the samples. Many of the studies (13/17) focused on self-reported surveys of SHS exposure not verified by nicotine biomarkers [8,23,26,27,30,31,32,33,36,37,38,39,40]. Among the four studies that did not implement surveys, Pitten et al. [34] and Pitten et al. [35] utilized an Automatic Environmental Tobacco Smoke Emitter to measure particulate matter (PM) concentrations and the effects of vehicle ventilation conditions. Continente et al. [28] collected air and dust samples to measure airborne nicotine concentrations. Jones et al. [29] measured SHS exposure by collecting biological samples of plasma cotinine and urine cotinine, 3-hydroxy cotinine (3HC), and 4-(methylnitrosamino)-(3-pyridyl)-1-butanol (NNAL).

Non-smokers exposed to SHS in vehicles showed significant increases in tobacco biomarkers (plasma cotinine and urine cotinine, 3-hydroxy cotinine (3HC), and urine 4-(methylnitrosamino-(3-pyridyl)1-butanol (NNAL)) shortly after exposure, which remained for 24 h, indicating the prolonged absorption and retention of SHS components [29]. Smokers who smoked inside a vehicle had median nicotine airborne concentrations of 3.5 μg/m^3^ (micrograms per cubic meter) compared to 0.23 μg/m^3^ for smokers who did not smoke inside their cars. Additionally, nicotine concentrations measured in the passenger compartment were six times higher during SHS exposure while traveling compared to concentrations in non-smokers’ cars, emphasizing the significant risk for non-smoker passengers in car cabins [28]. The studies conducted by Pitten et al. [34] and Pitten et al. [35] showed that even with ventilation, in-vehicle smoking generates harmful particle emissions. Concentrations were the highest when vehicle ventilation was turned off and all the windows were closed [34]. Opening the windows and using ventilation reduced PM concentrations but did not eliminate them [35]. The most effective ventilation condition was achieved by opening the vehicle window 10 cm with ventilation, creating an airstream rate of 25.1 to 25.8 km per hour at a 5 cm distance [35]. This condition lowered PM concentrations from 964–1697 µg/m^3^ to 68.9–139 µg/m^3^, still higher than the World Health Organization’s recommended limit (15 µg/m^3^) [41]. The research from the studies included in this review also shows that nearly one-third of United States youth are exposed to vehicular SHS exposure [26,30,33,37,38,39,40]. Specifically, female adolescents [26,30], non-Hispanic black people and non-Hispanic white people [38,40], non-urban youth [32], and those in socioeconomic areas [31] are more likely to experience SHS exposure in vehicles. Overall, these studies underscore the widespread prevalence of smoking in vehicles and the critical need for stringent SHS and THS exposure control.

## 4. Discussion

Results from this review yield no articles related to in-vehicle smoking among occupational and commercial drivers. The studies in this review predominantly focus on adolescents recognizing their vulnerability to SHS exposure due to their still-developing immune and respiratory systems [8]. However, it is crucial to acknowledge that adults, particularly non-smokers, are also significantly affected by both SHS and THS exposure, necessitating protection. This is especially relevant for occupational and commercial drivers, such as LHTDs, who experience prolonged exposures due to their unique work environments. Most critically, an LHTD who is a smoker receives nicotine exposure from all three sources: FHS, SHS, and THS. The critical distinction between nicotine exposures experienced by LHTDs and those of bus, cab, or taxi drivers is that LHTDs often live in their trucks for extended periods—days or even weeks at a time [42]. It has already been documented that tobacco smoke exposure in vehicles is associated with poor health outcomes, but prolonged times spent in a confined truck cab significantly increase the level of exposure, likely increasing the risk of lung cancer.

Despite its importance, nicotine exposure has primarily been studied using self-reporting methods to verify tobacco usage and exposure, which may lead to inaccurate measures of exposure. Robust measurement techniques, such as the use of nicotine biomarkers of smoking, should be used to quantify nicotine exposure and assess the associated health risks accurately [43]. This approach is essential for informing targeted interventions and policies to safeguard the health and well-being of vulnerable occupational groups, aligning with broader efforts to mitigate second-hand smoke exposure in vehicle environments and, ultimately, lung cancer risks.

Many workplaces have non-smoking policies and regulations, including bars and restaurants [13]. However, regulations only prohibit smoking in commercial trucks while they are hauling class 1 (explosives), 5 (oxidizer and organic peroxide), and flammable hazardous materials [44,45]. Smoking regulations in individual trucking companies and adherence to such policies are unknown [46]. Comprehensive laws and policies addressing smoking in vehicles should be established and enforced to help safeguard LHTDs. This targeted approach is essential for mitigating the health risks associated with tobacco smoke exposure in this unique occupational setting. To achieve this goal, it is imperative to conduct research toward the development of tailored smoking cessation interventions paired with incentives among LHTDs [2].

Although this review is primarily concentrated in the United States, occupational exposure to SHS and THS affects a wide variety of drivers globally, including taxi, cab, and ride-share drivers, who may also spend extended hours in poorly ventilated vehicles. Expanding research efforts to include diverse driver populations can serve as the foundation for generalizable policy development to protect these workers. While this review’s focus is situated on SHS, the global relevance of this issue also underscores the need for future research concerning THS exposure in vehicles.

## 5. Conclusions

The information provided in this narrative review is an in-depth exploration of tobacco smoke exposure, specifically in vehicles. This contributes significant insights into the multifaceted dimensions of smoke exposure and the subsequent gaps in the existing literature. Additionally, the text provides insights into the likelihood of elevated risks of tobacco smoke exposure among occupational and commercial drivers and the prevalence of smoking in this group. However, the state of science related to tobacco smoke exposures of occupational and commercial drivers is underdeveloped.

Indoor smoke elimination is identified as the primary preventive measure for non-smokers; however, the concentration of SHS exposure remains high in confined spaces like vehicles. The review highlights the presence of carcinogens, such as PAHs, at higher concentrations in vehicles compared to private settings, emphasizing the need for targeted interventions for workers who are exposed to tobacco smoke for long periods due to their work environment. This review’s findings underscore the urgent need for more research on tobacco smoke exposure in occupational and commercial vehicles. The review also emphasizes the need to develop targeted smoking cessation interventions, comprehensive legislation, and enforcement measures to safeguard public health and reduce or eliminate exposures, particularly among vulnerable populations such as workers in unique occupational environments like long-haul trucking. Future research should continue to explore the harmful effects of continuous smoking in enclosed spaces, providing a foundation for evidence-based policy and intervention strategies, potentially reducing healthcare costs associated with smoking-related diseases.

## Figures and Tables

**Table 1:** Terms Used to Search for Targeted Articles in PubMed, Scopus, and Embase.

Database	Search Terms
PubMed— Search conducted April 2025	(“Motor Vehicles”[Mesh] OR vehicle*[tiab] OR “Automobiles”[Mesh] OR automobile*[tiab] OR truck*[tiab] OR car[tiab] OR cars[tiab] OR SUV[tiab] OR SUVs[tiab] OR sports-utility-vehicle*[tiab]) AND (“Nicotine”[Mesh] OR nicotine[tiab] OR “Smoke/adverse effects”[Mesh] OR smoke-exposure*[tiab] OR secondhand-smoke*[tiab] OR thirdhand-smoke*[tiab] OR “Tobacco/adverse effects”[Mesh] OR “Tobacco Smoke Pollution”[Mesh] OR “Smoking, Passive”[Mesh] OR passive-smoking[tiab] OR involuntary-smoking[tiab] OR environmental-smoking[tiab])
Scopus— Search conducted April 2025	TITLE-ABS-KEY(“motor vehicle*” OR vehicle* OR automobile* OR truck* OR car OR cars OR suv OR suvs OR “sports utility vehicle*”) AND TITLE-ABS-KEY(nicotine OR “tobacco smoke pollution” OR “secondhand smoke” OR “thirdhand smoke” OR “passive smoking” OR “involuntary smoking” OR “environmental tobacco smoke” OR “smoke exposure” OR “tobacco smoke” OR “tobacco adverse effects” OR “smoke adverse effects”)
Embase— Search conducted April 2025	(‘motor vehicle’/exp OR ‘automobile’/exp OR ‘truck’/exp OR ‘car’/exp OR vehicle*:ti,ab OR automobile*:ti,ab OR truck*:ti,ab OR car:ti,ab OR cars:ti,ab OR suv:ti,ab OR suvs:ti,ab OR “sports utility vehicle*”:ti,ab) AND (‘tobacco smoke pollution’/exp OR ‘passive smoking’/exp OR ‘environmental tobacco smoke’/exp OR ‘involuntary smoking’/exp OR ‘secondhand smoke’/exp OR ‘thirdhand smoke’/exp OR ‘cigarette smoke’/exp OR ‘nicotine’/exp OR smoke exposure:ti,ab OR secondhand smoke:ti,ab OR thirdhand smoke:ti,ab OR passive smoking:ti,ab OR environmental tobacco smoke:ti,ab OR involuntary smoking:ti,ab OR nicotine:ti,ab OR smoke:ti,ab OR smoking:ti,ab) AND (‘driver’/exp OR ‘professional driver’/exp OR driver*:ti,ab OR truck*:ti,ab OR “long haul truck*”:ti,ab OR “commercial driver*”:ti,ab OR “occupational driver*”:ti,ab)

## Data Availability

This study is a review of the publicly available literature and does not involve the generation of new data. All sources used in the review are cited in the reference list. No additional data are available.

## Abbreviations

The following abbreviations are used in this manuscript:

LHTD	Long-haul truck drivers
FHS	First-hand smoke
SHS	Second-hand smoke
THS	Third-hand smoke
